# Headset-Type Biofluorometric Gas Sensor with CMOS for Transcutaneous Ethanol from the Ear Canal

**DOI:** 10.3390/s26092817

**Published:** 2026-04-30

**Authors:** Geng Zhang, Di Huang, Kenta Ichikawa, Kenta Iitani, Yoshikazu Nakajima, Kohji Mitsubayashi

**Affiliations:** 1 Laboratory for Biomaterials and Bioengineering, Institute of Integrated Research, Institute of Science Tokyo, 2-3-10 Kanda-Surugadai, Chiyoda-ku, Tokyo 101-0062, Japan; geng.z.0339@m.isct.ac.jp (G.Z.); ichikawa.kenta@tmd.ac.jp (K.I.); iitani.k.9009@m.isct.ac.jp (K.I.); nakajima@nakajimalab.org (Y.N.); 2 Graduate School of Medical and Dental Sciences, Institute of Science Tokyo, 1-5-45 Yushima, Bunkyo-ku, Tokyo 113-8510, Japan; hd1216@outlook.com

**Keywords:** wearable biosensor, biofluorometric detection, alcohol dehydrogenase, ear canal gas, non-invasive monitoring

## Abstract

This study presents a headset-type biofluorometric gas sensor incorporating a CMOS camera for continuous, non-invasive monitoring of transcutaneous ethanol from the ear canal. The sensor employs alcohol dehydrogenase (ADH) to catalyze the NAD^+^-to-NADH conversion during ethanol oxidation, enabling quantitative measurement through NADH fluorescence detection (λ_ex_ = 340 nm, λ_em_ = 490 nm). The integrated system comprises a wireless CMOS camera, an ADH-immobilized cotton mesh enzyme membrane, UV-LED excitation source, optical bandpass filters, and a dual convex lens assembly housed in a 3D-printed headset powered by a lithium battery. Key improvements include a 3.5-fold enhancement in fluorescence collection efficiency achieved through optimized dual convex lens configuration. Systematic screening of seven cotton mesh materials identified Iwatsuki cotton mesh as the optimal enzyme immobilization substrate, exhibiting minimal autofluorescence and 14.2-fold higher water retention capacity compared to H-PTFE membranes. The glutaraldehyde-crosslinked ADH-immobilized cotton mesh maintained enzymatic activity for over 45 min with a 10-fold improvement in signal-to-noise ratio. The system demonstrated a dynamic detection range spanning 10 ppb to 10 ppm for gaseous ethanol and exhibited high selectivity against interfering volatile organic compounds in skin gas, including methanol, acetaldehyde, formaldehyde, and acetone. Human experiments validated the system’s practical performance. Following alcohol consumption, subjects wore the device for 50 min while real-time fluorescence monitoring captured dynamic ethanol concentration changes in the ear canal. The dose-dependent fluorescence response—approximately 2-fold higher at 0.4 g/kg versus 0.04 g/kg alcohol intake—correlated well with calibration data. This headset-type biofluorometric sensor enables unrestrained continuous monitoring of ear canal ethanol, providing a novel wearable platform for alcohol metabolism assessment with potential applications in health monitoring and clinical research.

## 1. Introduction

Human volatile organic compounds (VOCs) are gaseous byproducts generated during metabolic processes. They are widely present in exhaled breath, skin-released gases, and body cavity gases. The types and concentration changes of these VOCs are closely related to specific physiological and pathological states in the body. For example, acetone is associated with diabetic ketoacidosis metabolism [1,2,3], carbon monoxide with asthma [4], and sulfur-containing compounds with liver diseases [5,6]. Therefore, non-invasive detection of human VOCs has received widespread attention in recent years as a potential tool for disease diagnosis and health monitoring.

Among various human VOCs, ethanol is one of the most representative and clinically valuable targets. Under normal physiological conditions, the endogenous ethanol concentration in human blood is below 1.6 μg/mL (typically 0–0.8 μg/mL). The corresponding exhaled ethanol concentration is approximately 2–10 ppb [7,8]. After alcohol consumption, ethanol is rapidly absorbed into the bloodstream through the stomach and small intestine. It can be detected in blood about 5 min later and reaches peak concentration within 30–90 min [9]. About 90% of ethanol in the body is metabolized to acetaldehyde by alcohol dehydrogenase (ADH) in the liver. This process is accompanied by the conversion of coenzyme NAD^+^ to NADH. Acetaldehyde is then further oxidized by aldehyde dehydrogenase (ALDH) [10]. It is finally excreted through urine, exhaled breath, and sweat [11]. Blood alcohol concentration (BAC) is the primary quantitative indicator used in legal and medical contexts for assessing the level of alcohol intoxication. There is an approximate 2100:1 conversion ratio between BAC and exhaled ethanol concentration [12]. Therefore, ethanol vapor released through transcutaneous or respiratory routes can indirectly reflect BAC. It has important application prospects in alcohol metabolism kinetics assessment, real-time monitoring of drinking status, clinical management of alcohol use disorders, and liver function screening. Current common methods for ethanol detection include blood sampling, urine analysis, and breath testing [13]. Although blood testing has the highest accuracy, it is an invasive procedure and inconvenient for sampling. Urine tests are commonly used to detect the presence of ethanol in a sample, but it is difficult to quantify the concentration of ethanol. Breath testing has the advantages of being non-invasive and real-time, but it requires active cooperation from subjects for breathing operations. It is not suitable for long-term passive monitoring in uncontrolled scenarios such as sleep or exercise [14]. Therefore, there is an important practical need to develop a non-invasive technology that can continuously detect ethanol vapor without requiring active cooperation from subjects.

Regarding VOC gas analysis technologies, current methods mainly include gas chromatography-mass spectrometry (GC-MS), proton transfer reaction mass spectrometry (PTR-MS), selected ion flow tube mass spectrometry (SIFT-MS), semiconductor metal oxide sensors, and electronic noses [15,16,17,18]. Although these large analytical instruments have high sensitivity and good VOC component identification capabilities, they are bulky and complex to operate. They are difficult to meet the needs for real-time continuous monitoring of transcutaneous VOCs. In terms of sensors, Lansdorp et al. [19] developed a wristband-type wearable alcohol sensor based on alcohol oxidase and Prussian blue. It uses electrochemical methods to detect transcutaneous ethanol. Kim et al. reported a wearable alcohol monitoring device based on a tattoo-type iontophoresis-biosensing system [20]. However, the above sensors still have deficiencies in selectivity and detection capability for low-concentration transcutaneous VOCs. They struggle to simultaneously achieve high sensitivity, high selectivity, and dynamic continuous measurement.

To address the above issues, our research group previously developed a biofluorometric gas sensing system (bio-sniffer/sniff-cam) that uses enzymatic reactions as molecular recognition elements. This system is based on the interconversion between NAD^+^ and NADH during the oxidation (or reduction) of target VOCs catalyzed by NADH-dependent dehydrogenases. It achieves quantitative measurement of target gases by detecting the autofluorescence signal of NADH at 490 nm under 340 nm excitation. Compared with traditional physicochemical sensors, enzyme sensors have molecular-level substrate selectivity. The fluorescence detection method provides high sensitivity. Based on this platform, our group has successfully achieved highly selective detection of various VOCs including ethanol, methanol, acetone, isopropanol, formaldehyde and 2-nonenal.

Current wearable alcohol monitoring technologies predominantly rely on liquid-phase detection of ethanol from body fluids rather than gas-phase measurement. Fairbairn et al. reported a large-scale validation study of transdermal alcohol sensors that detect ethanol vapor emitted through perspiration and insensible perspiration from skin surface, achieving strong accuracy for real-time drinking detection [21]. However, these transdermal sensors measure alcohol diffused through sweat and skin capillaries, which involve complex lag times and environmental confounds from perspiration rate and skin-sensor distance. Similarly, most existing wearable alcohol monitors are based on electrochemical detection of alcohol in sweat or interstitial fluid, limiting their application for direct gas-phase monitoring. For ear canal gas monitoring, Mo et al. [22] recently developed a wearable SPME-in-earmuffs device for sampling volatile organic compounds from human ears. Their method successfully extracted various metabolites, environmental exposures from the ear canal. However, this approach requires offline GC-MS analysis after SPME sampling, preventing continuous monitoring. Therefore, a wearable system capable of direct, real-time gas-phase detection of ethanol vapor in the ear canal has not yet been achieved.

Regarding sampling sites, transcutaneous gas (skin gas) is more suitable for long-term continuous monitoring compared to exhaled breath. This is because its release is unconscious and continuous, with fewer restrictions on time and location. However, transcutaneous VOC detection faces two major challenges. First, transcutaneous gas concentration is much lower than exhaled breath [23,24]. Second, there are sweat interferences and differences in release kinetics at different body surface sites due to varying sweat gland densities and epidermal thicknesses [25,26]. Therefore, selecting a body surface area with relatively high VOC concentration and minimal sweat interference is crucial for transcutaneous VOC monitoring.

Recent research has found that the external auditory canal (EAC) and external ear area have unique anatomical advantages. The sweat gland density in the external ear area is much lower than that of the palm, forearm, and cheek [27]. Research by Lobitz et al. further showed that there are no eccrine sweat glands in the ear canal [28]. In addition, gases in the ear canal not only originate from transcutaneous release from external ear skin but may also contain VOCs that permeate from the middle ear cavity through the tympanic membrane [29,30]. The middle ear mastoid air cells have thin squamous epithelium similar to alveoli and a rich capillary network. This facilitates the permeation of gaseous components from the blood [31,32]. These characteristics make the VOC concentration in the external ear area significantly higher than other body surface sites, while sweat interference with sensing signals is minimal [22].

Based on this background, our research group has conducted a series of studies on external ear VOC detection. Toma et al. first developed an external ear ethanol monitoring system using an over-ear gas collection chamber and fiber-optic biosniffer, though its reliance on benchtop instruments prevented wearable applications [33]. To achieve unrestrained measurement, Arakawa et al. subsequently integrated an ADH enzyme membrane, UV-LED, and wireless CMOS camera into a battery-powered headset system, successfully monitoring metabolic changes in ear canal ethanol during human drinking experiments [34]. Recently, we extended this platform to acetone detection using S-ADH enzyme reactions, verifying good correlation between ear canal and exhaled acetone, further demonstrating the platform’s versatility [35].

Although previous research demonstrated the feasibility of ear canal VOC monitoring, existing systems have limitations: the CMOS camera approach shows lower sensitivity and signal-to-noise ratio than fiber-PMT systems, enzyme membrane stability requires optimization, and no integrated solution combines high sensitivity with wearable portability. To address these issues, this study introduces three innovations: (1) cotton mesh as a novel enzyme immobilization matrix with improved water retention capacity; (2) dual convex lens optical architecture for enhanced light collection efficiency; and (3) differential fluorescence analysis for quantitative kinetic monitoring. These advances enable a wearable biosniffer achieving high sensitivity, extended operation time, and quantitative tracking of ear canal ethanol metabolism in humans.

## 2. Experiment

### 2.1. System Construction and Sensing Principle

**Figure 1:**
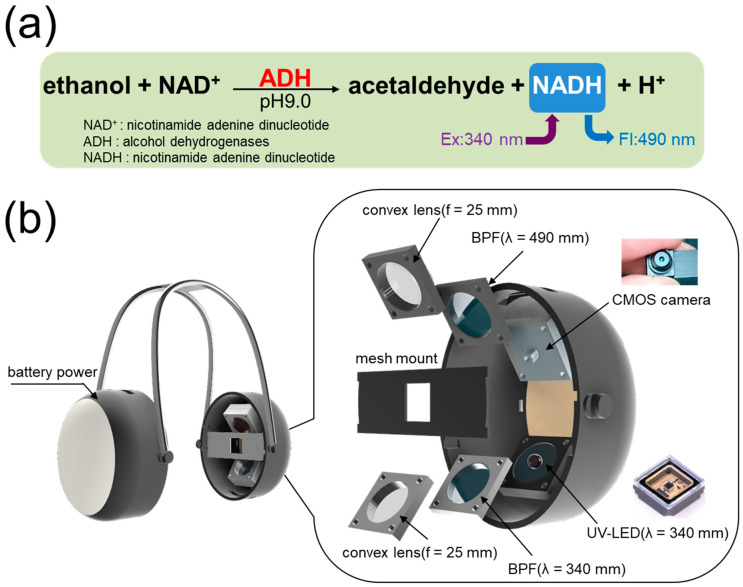
(**a**) Measurement principle of gaseous EtOH based on biofluorometry. (**b**) Design and component layout of the headset-type biofluorometric gas sensor.

Gaseous EtOH was selected as the target analyte. The EtOH sensing principle, based on NAD^+^-dependent ADH (EC 1.1.1.1), is illustrated in Figure 1a. EtOH is catalyzed by ADH in the presence of the oxidized form of NAD (NAD^+^) at pH 9.0, producing acetaldehyde and the reduced form of NAD (NADH). NADH exhibits fluorescence at 490 nm upon UV excitation at 340 nm, and its fluorescence intensity is proportional to its concentration. Since the amount of NADH produced correlates directly with the EtOH concentration, quantification of gaseous EtOH is possible by measuring the NADH fluorescence intensity. Figure 1b shows the design of the headset-type biosniffer. The left ear cup contains a rechargeable lithium-ion battery (product# P11-18650STD-D, 3.7 V, 2500 mAh, SEnergy Co., Kanagawa, Japan) with a dedicated lithium-ion battery charging circuit (product# LM3658SD-AEV, Texas Instruments, Dallas, TX, USA) for powering the biofluorometric gas sensing unit implemented in the right ear cup. The optical sensing unit comprises a UV-LED (product# 340-FL-01-G01, λ = 340 nm, max power: 40 mW, adjustable applied current up to 100 mA, DOWA Electronics, Tokyo, Japan), a 340 nm bandpass filter for excitation light (BPFex, product# MX0340, λ = 340 ± 10 nm, Asahi Spectra, Tokyo, Japan), a 490 nm bandpass filter for fluorescence detection (BPFfl, product# MX0490, λ = 490 ± 10 nm, Asahi Spectra, Tokyo, Japan), and a Wi-Fi CMOS camera (product# k-camera-020, frame rate 30 fps, maximum resolution 2K, zoom ratio 1:1, D-SUPLLY, Shenzhen, China). The Wi-Fi CMOS camera and the UV-LED were positioned 30 mm and 35 mm from the ADH-immobilized mesh, at angles of 48° and 42° with respect to the mesh surface, respectively. The device housing was designed in 3D CAD software (Fusion 360, version 2.0, Autodesk, San Francisco, CA, USA) and fabricated by a 3D printer (product# Form 3B, resin-based SLA, Formlabs, Somerville, MA, USA). All components were assembled without adhesive materials to avoid unwanted interference from EtOH released by adhesives.

### 2.2. Optimization of Optical Components and Immobilization Substrate

#### 2.2.1. Convex Lens Configuration

The possibility of sensitivity improvement through enhanced light collection efficiency was evaluated by employing two plano-convex lenses (product# SLB-25B-25PM, f = 25 mm, diameter: 25 mm, OptoSigma, Tokyo, Japan). One lens was placed in front of the camera to adjust the focal length, and the other was placed in front of the UV-LED to concentrate the excitation light onto the sensing surface. The effect of the dual lens configuration was assessed by comparing the resultant fluorescence intensity from a cotton mesh test piece (product# 13700000, Orange Care, Osaka, Japan) with and without the lenses.

#### 2.2.2. Selection of Enzyme Immobilization Substrate

In order to extend the usable time of the system, the water retention ability of the enzyme immobilization substrate was considered a critical factor. The water imbibition capacity and evaporation rate of the hydrophilic PTFE membrane (H-PTFE, product# JGWP04700, pore size: 0.2 μm, thickness: 47 μm, Merck Millipore, County Cork, Ireland)—used in the previously developed system—and cotton mesh were compared using a calibrated balance.

Subsequently, autofluorescence—which determines the background level of the detection system—was compared among seven types of cotton mesh from different manufacturers (#1: Matsumae, #2: Suzurann, #3: Iwatsuki, #4: Daiso, #5: Shiai, #6: Hakujuji, and #7: Askul) and H-PTFE membrane. All substrates were evaluated under the same optical setup in both dry and wet conditions.

### 2.3. Enzyme Immobilization

An ADH-immobilized mesh was prepared using the GA cross-linking method. First, 0.44 mg of alcohol dehydrogenase (ADH, product# A7011, lyophilized powder, 128 units/mg, Sigma-Aldrich, St. Louis, MO, USA) and 5.6 mg of bovine serum albumin (BSA, product# A2153, Wako, Tokyo, Japan) were dissolved in phosphate buffer (PB, pH 8.0, 100 mM, 112.5 μL). Second, 112.5 μL of the ADH solution was dropped onto a 15 × 15 mm^2^ cotton mesh and refrigerated at 4 °C for 60 min. Third, 18 μL of glutaraldehyde solution (2.5 *v/v*%, 100 mM, pH 8.0, diluted with PB) was applied to the ADH-absorbed mesh and refrigerated at 4 °C for 90 min. The mesh was then rinsed with Tris-HCl buffer (100 mM, pH 9.0, 500 μL) to remove non-immobilized ADH. In previous studies, ADH-immobilized membranes were prepared by coating a mixture of 40 µL ADH enzyme solution (128 units/mg) and 40 µL poly[MPC-co-EHMA] (PMEH, 15 wt% in ethanol) onto hydrophilic porous PTFE membrane (pore size 0.2 µm, thickness 65 µm) to achieve 60 units/cm^2^, followed by drying at 4 °C for 3 h. Before each measurement, the ADH-immobilized mesh was wet with 80 μL of NAD^+^ solution (10 mM in 100 mM Tris-HCl, pH 9.0).

### 2.4. Performance Evaluation with Standard EtOH Gas

The ADH-immobilized mesh was placed at the biofluorometric gas sensing unit of the system. Dry clean carrier air was supplied to a standard gas generator (product# PD-1B-2, Gastec, Kanagawa, Japan) via a PTFE tube (product# f-8006-017, inner diameter: 4 mm, FLON Industry, Tokyo, Japan). Known concentrations of EtOH vapor generated by the gas generator and dry clean carrier air were selectively applied to the ADH-immobilized mesh via a solenoid valve system (product# VX-140H, 24 V DC, SMC, Tokyo, Japan) controlled by a single-chip microcomputer (Arduino DUE). The gas outlet was directed at the center of the ADH-immobilized mesh, and standard EtOH gas was applied according to the following protocol: (I) carrier air for 120 s; (II) standard EtOH gas at 100 mL/min for 120 s; (III) return to carrier air for 180 s.

Changes in fluorescence intensity on the ADH-immobilized mesh were recorded by the Wi-Fi CMOS camera. Since the NADH fluorescence at 490 nm is detected in both the green and blue channels of the Bayer-filtered CMOS sensor, the blue channel gray value was used to quantify NADH fluorescence intensity. The image processing workflow is illustrated in Figure S1, showing the original color image, blue channel extraction, and differential image analysis. The central circular region marked by the red dashed line indicates the gas delivery area on the ADH-immobilized membrane surface. Captured images were analyzed using image processing software (ImageJ, version1.54r) with a custom Python (version 3.13.x) script to calculate the fluorescence change rate. To determine the dynamic range, standard EtOH gas at concentrations of 0.01, 0.05, 0.1, 0.5, 1, 3, 10, 30, 100, 300, and 500 ppm was measured. In addition, NAD^+^ loading and the selectivity of the system against EtOH were evaluated by testing typical H-VOCs (methanol, acetaldehyde, formaldehyde, 1-butanol, 1-propanol, 2-propanol, and acetone) at 1 ppm each.

A differential analysis was applied to the fluorescence time-course data to extract the real-time rate of fluorescence change per unit time, according to the following equation:

(1) dI/dt=[g(t+Δt)−g(t)]/Δt

where *I* is the fluorescence intensity (a.u.), *t* is the time (s), Δt is the time interval (s), and g(t) is the fluorescence intensity at time t.

### 2.5. Long-Term Ear Canal EtOH Gas Monitoring

Since transdermal gas is emitted by diffusion rather than convective flow, the gas outlet was covered with a PTFE membrane (product# WP-500-100, pore size: 5 μm, thickness: 100 μm, Sumitomo Electric Fine Polymer, Osaka, Japan) to mimic gas diffusion through the skin in the long-term measurement evaluation. The flow rate of both carrier air and standard EtOH gas was set to 1 mL/min. In the experiment, 35 min of EtOH gas was applied after a 10-min carrier air baseline. EtOH concentrations of 50, 100, 200, 500, and 1000 ppb were used to determine a calibration curve for ear canal EtOH.

### 2.6. Human Subject Experiment

The improved headset-type biosniffer was used to demonstrate continuous monitoring of alcohol metabolism in a healthy human subject. One healthy male subject (age: 28 years) participated in the study with informed consent. This study was approved by the Institutional Review Board of the School of Medicine, Institute of Science Tokyo (Authorization Number: M2018-160). The subject was tested at two alcohol dosages (0.04 g/kg and 0.4 g/kg body weight) on different days to evaluate dose-dependent response. The subjects were informed of the purpose and significance of the experiment by written documentation from the study conductor and provided with written informed consent. Prior to the experiment, an alcohol patch test was performed to briefly classify the aldehyde dehydrogenase type 2 (ALDH2) phenotype, which is related to alcohol metabolism. Body temperature, blood pressure, and heart rate were also recorded alongside a lifestyle questionnaire. Subjects were asked to avoid alcoholic beverages and medication for 72 h and meals for 4 h prior to the experiment.

In the experiment, subjects consumed a normalized amount of alcoholic beverage (0.4 g/kg body weight) within 15 min. They were then asked to rinse their mouths with 100 mL of water to remove residual alcohol from the oral cavity. Immediately afterward, the headset-type biosniffer was worn by the subject, and ear canal EtOH was monitored continuously for up to 50 min.

All experiments were performed in a climate-controlled laboratory maintained at 25 °C with humidity monitoring. Although humidity was not actively controlled, the stable indoor environment minimized fluctuations in environmental conditions during measurements.

## 3. Results and Discussion

### 3.1. Optimization of Optical Components

Figure S2 shows the fluorescence images from a cotton mesh test piece captured without and with convex lenses, along with the corresponding fluorescence intensities. The fluorescence intensity from the test piece increased 3.5-fold when convex lenses were employed, owing to the stronger UV excitation light focused onto the sensing surface by the lens in front of the UV-LED and the improved fluorescence collection by the lens in front of the camera. This enhancement is expected to directly contribute to improved detection sensitivity for low-concentration ear canal EtOH. Accordingly, convex lenses were used in all subsequent experiments.

### 3.2. Selection Result of Enzyme Immobilization Substrate

Figure S3a shows the comparison of water imbibition between the H-PTFE membrane and the cotton mesh. The cotton mesh exhibited 14.2-fold higher water imbibition capacity than H-PTFE, indicating significantly better liquid absorption—an important property for maintaining enzyme activity during long-term experiments. Furthermore, a slower water evaporation rate was observed for the cotton mesh compared to H-PTFE (Figure S3b): H-PTFE dried completely within approximately 25 min, while the cotton mesh continued to evaporate slowly over more than 40 min. Based on these results, cotton mesh was selected as the immobilization substrate for subsequent experiments.

**Figure 2:**
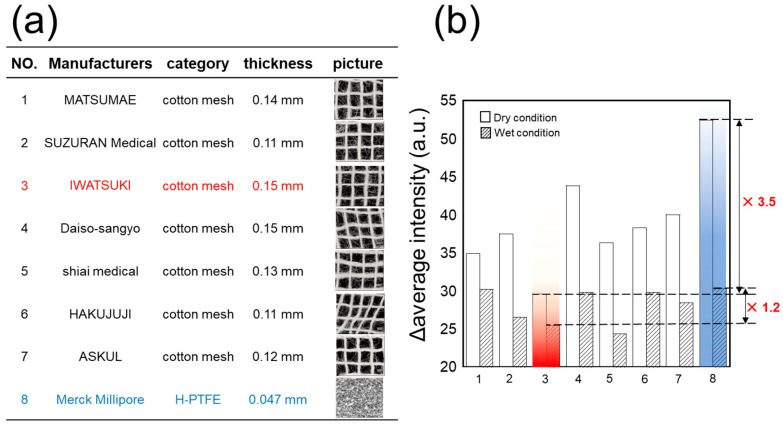
(**a**) Specification table of seven types of cotton mesh and H-PTFE membrane evaluated as enzyme immobilization substrates. (**b**) Comparison of autofluorescence (Δaverage intensity, a.u.) under dry and wet conditions. The red and blue bars highlight the autofluorescence of the selected optimal substrate (No. 3, Iwatsuki cotton mesh) and the H-PTFE membrane (No. 8, Merck Millipore) under dry and wet conditions, respectively.

Figure 2 shows the comparison of autofluorescence among the seven cotton meshes and H-PTFE membrane in dry and wet conditions. Lower autofluorescence was observed in wet conditions compared to the dry condition in all samples. Among the seven cotton meshes, the #3 Iwatsuki cotton mesh showed the lowest autofluorescence. The #3 mesh exhibited 3.5-fold and 1.2-fold lower autofluorescence than the H-PTFE membrane under dry and wet conditions, respectively. Lower autofluorescence directly contributes to higher sensitivity by increasing the signal-to-background ratio; accordingly, the #3 Iwatsuki cotton mesh was selected as the immobilization substrate for ADH.

### 3.3. Comparison of Enzyme Immobilization Methods

**Figure 3:**
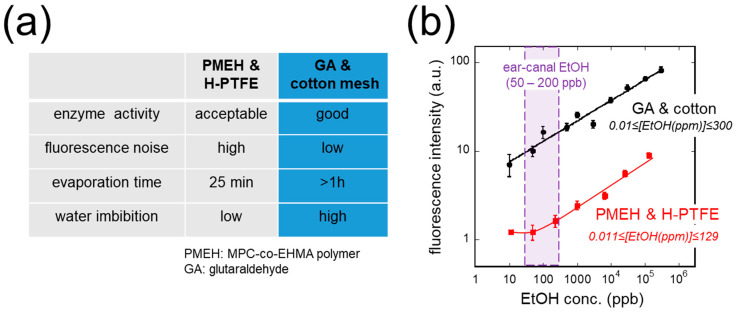
Comparison of two enzyme immobilization methods. (**a**) Summary of enzyme activity, fluorescence noise, evaporation time, and water imbibition for PMEH & H-PTFE and GA & cotton mesh methods. (**b**) Calibration curves on a log-log scale.

Figure 3 presents the comparison between the PMEH & H-PTFE and GA & cotton mesh immobilization methods. As summarized in Figure 3a, the GA & cotton mesh method demonstrated superior performance in all evaluated metrics: better enzyme activity, lower fluorescence noise, longer water evaporation time (>1 h vs. 25 min), and higher water imbibition capacity.

The calibration curves in Figure 3b show that the GA & cotton mesh method achieved a quantification range of 0.01–300 ppm [EtOH], with approximately 10-fold higher fluorescence output than the PMEH & H-PTFE method (0.011–129 ppm [EtOH]) across the entire concentration range. The signal-to-noise ratio of the GA & cotton mesh method was 10 times greater. The expected ear canal EtOH concentration range after drinking (50–200 ppb, shaded region) is fully covered by the GA & cotton mesh quantification range. Based on these results, the GA & cotton mesh method was adopted for all subsequent experiments.

### 3.4. Basic Characteristics of the Headset-Type Biosniffer

**Figure 4:**
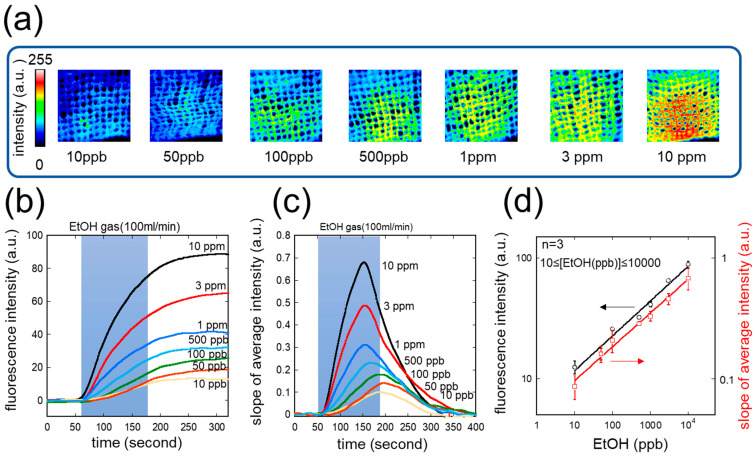
Basic characteristics of the headset-type biosniffer with standard EtOH gas (10 ppb to 10 ppm). (**a**) False-color fluorescence images of the ADH-immobilized mesh at 10 ppb, 50 ppb, 100 ppb, 500 ppb, 1 ppm, 3 ppm, and 10 ppm. (**b**) Time courses of fluorescence intensity at various EtOH concentrations. Shaded area: EtOH gas loading period (100 mL/min). (**c**) Time courses of the slope of average fluorescence intensity from differential analysis. (**d**) Calibration curves from stable fluorescence intensity (black circles, left axis) and peak differential signal (red squares, right axis) on a log-log scale. Error bars represent standard deviation (*n* = 3). Some error bars are smaller than the symbol size due to high reproducibility.

Figure 4 shows the response of the headset-type biosniffer to standard EtOH gas at concentrations ranging from 10 ppb to 10 ppm. As shown in Figure 4a, fluorescence images of the ADH-immobilized mesh clearly illustrate the concentration-dependent increase in NADH fluorescence: the false-color images progress from deep blue at 10 ppb to yellow green at 10 ppm, providing a visually direct readout of EtOH concentration across seven concentration levels.

The time courses of fluorescence intensity (Figure 4b) show that the signal increases rapidly upon EtOH gas loading (shaded region) and reaches a stable value after gas loading is stopped, consistent with the irreversible accumulation of NADH on the enzyme membrane. The stable fluorescence values were clearly distinguishable across all tested concentrations from 10 ppb to 10 ppm.

In addition to the cumulative fluorescence signal, differential images of the fluorescence enabled real-time visualization of the rate of change in gaseous EtOH concentration on the ADH-immobilized mesh. The slope of fluorescence intensity represents the NADH generation rate—i.e., the rate of the ADH-mediated reaction—thereby enabling real-time monitoring of gaseous EtOH concentration (Figure 4c). A sharp, concentration-dependent peak was observed during the gas loading period, with the signal returning to baseline after gas loading stopped.

Calibration curves derived from the stable fluorescence intensity and the peak differential signal are shown in Figure 4d. Each calibration point represents the meaning of three independent measurements (*n* = 3) using freshly prepared ADH-immobilized membranes. Both showed good log-log linearity over the range of 10 ppb to 10 ppm (*n* = 3). The limit of detection (LOD), calculated as 3σ/slope from the calibration curve, was 1.5 ppb, and the limit of quantification (LOQ, 10σ/slope) was 4.9 ppb. The power-law fitting equations are as follows:

Fluorescence intensity = A × EtOH conc. [ppb]^B(2)

where A = 4.4, B = 0.23

Slope of average intensity = A × EtOH conc. [ppb]^B(3)

where A = 3.6, B = 0.27

The dynamic range of the differential signal-based calibration (Equation (3)) encompasses the expected ear canal EtOH concentration range after alcohol ingestion [34], confirming that quantitative monitoring of ear canal EtOH is feasible with the improved headset-type biosniffer.

### 3.5. Selectivity

**Figure 5:**
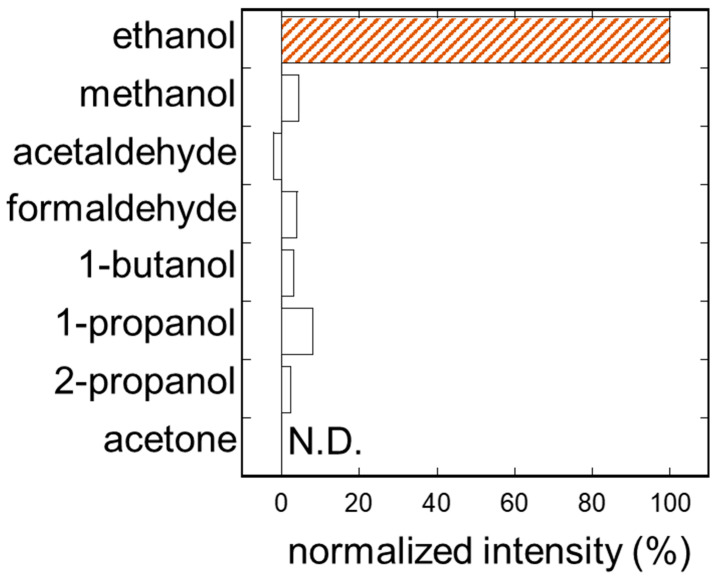
Selectivity of the headset-type biosniffer against typical VOCs in human skin gas. Each gas was tested at 1 ppm and the response was normalized to that of ethanol (100%). The orange hatched bar indicates the target analyte (ethanol).

Figure 5 shows the relative output to typical VOCs in skin gas at 1 ppm, compared to that of EtOH. Relatively low signals were observed for all tested species. Methanol and 1-propanol showed cross-responses of approximately 5% and 10% relative to EtOH, respectively, while acetaldehyde, formaldehyde, 1-butanol, 2-propanol, and acetone produced negligible signals (acetone: N.D.). These low cross-responses are attributed to the high substrate specificity of ADH from Saccharomyces cerevisiae toward primary alcohols. These results suggest that this human experiment represents a proof-of-concept demonstration with a single subject system that can accurately measure EtOH concentration without significant interference from other VOCs present in skin gas.

### 3.6. Long-Term EtOH Gas Loading

Figure S4a shows the time course of fluorescence intensity obtained by continuous application of standard EtOH gas for 35 min (1 mL/min). The fluorescence continued to increase at an almost constant rate and then saturated in order of higher concentration. The time until saturation was approximately 20 min at 1 ppm—the highest concentration tested. The saturated signal levels were approximately constant even when the EtOH concentration was varied, suggesting that the limiting factor is the depletion of NAD^+^ at the end of the experiment. Therefore, the usable time can potentially be extended by increasing the amount of pre-loaded NAD^+^ on the enzyme membrane.

The differential signals from this experiment are shown in Figure S4b. The maximum values of the differential signal followed the EtOH concentration in a manner consistent with the short-term EtOH application results. The calibration curve for long-term monitoring of ear canal EtOH is shown in Figure 5 and was calculated from the peak maximum of the differential signal at 0.05–1 ppm. The fitting equation (Equation (4)) yielded a correlation coefficient of 0.97:

Slope of average intensity = A × EtOH conc. [ppb]^B(4)

where A = 1.42, B = 0.26

These results demonstrate that the differential analysis approach enables quantitative long-term dynamic monitoring of ear canal EtOH gas, with an estimated optimal measurement window of within 1.5 h.

### 3.7. The Result of Human Subject Experiment

**Figure 6:**
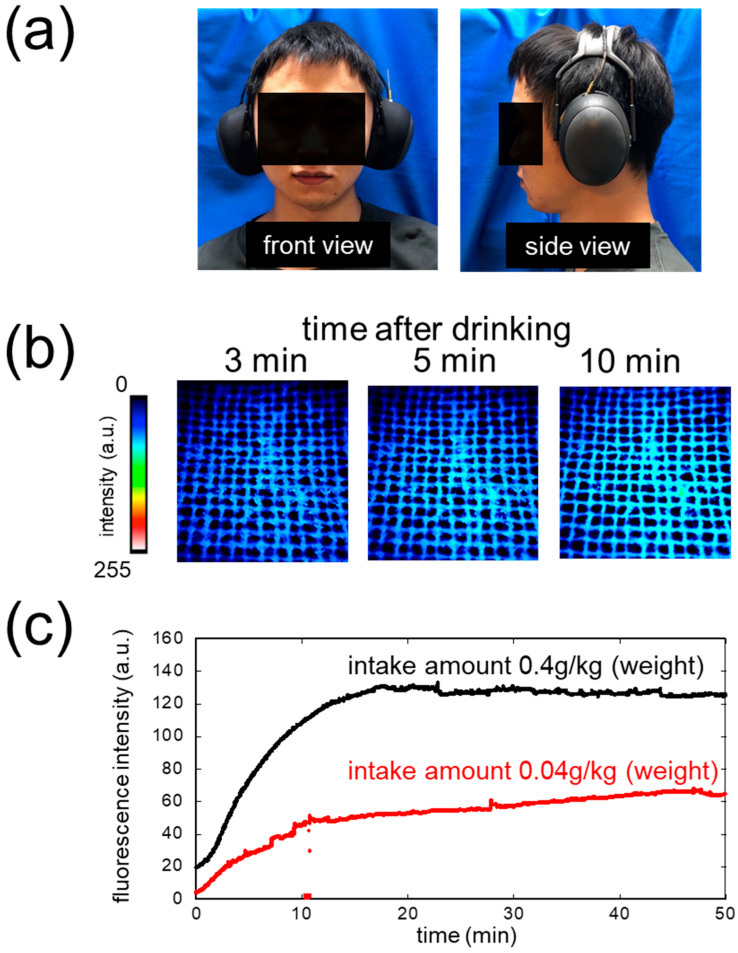
Human subject experiment. (**a**) Front and side view photographs of the subject wearing the headset-type biosniffer. (**b**) False-color fluorescence images of the ADH-immobilized mesh at 3, 5, and 10 min after alcohol ingestion (0.04 g/kg body weight). (**c**) Time courses of fluorescence intensity for two alcohol intake amounts (0.4 g/kg, black; 0.04 g/kg, red) recorded over 50 min.

Figure 6a shows the front and side views of a subject wearing the headset-type biosniffer. By employing the wireless fluorescence measurement system, the subject was able to move freely and comfortably during the long-term experiment.

Figure 6b shows false-color fluorescence images of the ADH-immobilized mesh captured at 3, 5, and 10 min after alcohol ingestion (0.04 g/kg body weight). A progressive increase in NADH fluorescence intensity was observed over time, directly confirming that ethanol vapor released from the ear canal was captured and detected in real time by the wearable biosniffer.

Figure 6c shows the time courses of fluorescence intensity after alcohol administration for two intake amounts. The fluorescence signal increased immediately after the subject wore the biosniffer, which disturbed the acquisition of a stable baseline for the differential signal. The ear canal and oral cavities are internally connected via the Eustachian tube. Although exhaled air is not directly vented into the ear canal due to the presence of the tympanic membrane, diffusion of low-concentration exhaled EtOH gas through the tympanic membrane into the ear canal is possible—this is considered to be the cause of the initial rise in fluorescence. Additionally, fluorescence saturation was reached faster (approximately 10 min) in the human subject experiment than in the standard gas experiment (approximately 20 min). This phenomenon is attributed to unknown environmental effects within the sealed ear cup, including possible increases in local temperature and humidity from the human subject, and warrants further investigation. A critical limitation is the inability to distinguish true ear canal ethanol signals from device artifacts. The immediate fluorescence increase prevents stable baseline establishment, and we cannot determine whether signals originate from transcutaneous ethanol, exhaled ethanol permeating through the tympanic membrane, or environmental factors (temperature, humidity) within the sealed ear cup. This mechanistic ambiguity must be resolved before clinical use. Future studies will include temperature/humidity monitoring, sealed versus vented configurations, and simultaneous breath measurements to isolate these factors.

Despite these differences between the standard gas and human subject experiments, clear dose-dependent fluorescence responses were observed between the 0.04 g/kg and 0.4 g/kg intake conditions. The fluorescence response for the 0.4 g/kg condition (~130 a.u.) was approximately twice that of the 0.04 g/kg condition (~65 a.u.), consistent with the calibration data. Both curves plateaued within approximately 20–30 min, reflecting the kinetics of alcohol absorption and distribution. Limitations include lack of 0 g/kg control and BAC comparison. Future studies will establish quantitative correlation with blood alcohol. This proof-of-concept demonstrates the wearable platform’s dose-dependent monitoring capability.

## 4. Conclusions

In this study, a headset-type biofluorometric biosniffer was developed and evaluated for continuous, non-invasive monitoring of ear canal EtOH vapor. The system was constructed using a wireless CMOS camera, an ADH-immobilized cotton mesh enzyme membrane, a UV-LED, bandpass filters, and convex lenses. The newly designed dual convex lens optical system improved excitation and fluorescence light collection efficiency by 3.5-fold. The highly moistened ADH-immobilized cotton mesh enabled maintenance of enzyme activity for longer periods (>45 min) compared to the previously used ADH-immobilized H-PTFE membrane (<5 min). The system demonstrated a wide dynamic range for gaseous EtOH measurement (10 ppb to 10 ppm) and high selectivity for EtOH against other VOCs in skin gas. Furthermore, consistent fluorescence signals were observed at concentrations relevant to ear canal EtOH after alcohol ingestion (50–1000 ppb). In the human subject experiment, dynamic changes in fluorescence intensity reflecting alcohol absorption and metabolism were successfully monitored in real time using the wearable headset-type device. The observed dose-dependent response demonstrates the technical feasibility of the platform, though clinical validation requires larger-scale studies. These results establish the proof-of-concept of the headset-type biosniffer as a platform for continuous, non-invasive alcohol metabolism monitoring, with potential future applications in health monitoring and clinical research after further validation.

## Data Availability

The data presented in this study are available on request from the corresponding author.

## Supplementary Materials

The following supporting information can be downloaded at: https://www.mdpi.com/article/10.3390/s26092817/s1, Figure S1: Representative fluorescence images with labeled key regions demonstrating the image processing workflow for NADH fluorescence quantification, including original color image, blue channel extraction, and differential image analysis. The central circular region (marked by red dashed line) indicates the gas delivery area on the ADH-immobilized membrane surface. Figure S2: Effect of convex lens configuration on fluorescence detection efficiency. (a) Fluorescence images of a cotton mesh test piece excited and captured without (w/o lens) and with convex lenses (w/lens). (b) Comparison of average fluorescence intensity. Figure S3: Water retention properties of H-PTFE membrane and cotton mesh. (a) Water imbibition capacity. (b) Water evaporation rate. Figure S4: Long-term EtOH gas loading results. (a) Time courses of fluorescence intensity at EtOH concentrations of 50 ppb, 100 ppb, 200 ppb, 500 ppb, and 1 ppm. (b) Time courses of the slope of average fluorescence intensity. Figure S5: Calibration curve for long-term monitoring of ear canal EtOH.
