# Supplementary material for: Headset-Type Biofluorometric Gas Sensor with CMOS for Transcutaneous Ethanol from the Ear Canal
Source: Sensors (Basel). 2026 Apr 30;26(9):2817. doi: 10.3390/s26092817 (PMC13165704; doi:10.3390/s26092817)
Supplement: Supplementary file 1 [file sensors-26-02817-s001.zip › sensors-4273928-supplementary.pdf]

Supporting Information for

Headset-Type Biofluorometric Gas Sensor with CMOS for Transcutaneous Ethanol from the Ear Canal

Geng Zhang [1], Di Huang [2], Kenta Ichikawa [1], Kenta Iitani [1], Yoshikazu Nakajima [1] and Kohji Mitsubayashi [1,*]

[1] Laboratory for Biomaterials and Bioengineering, Institute of Integrated Research, Institute of Science Tokyo, 2-3-10 Kanda-Surugadai, Chiyoda-ku, Tokyo 101-0062, Japan; geng.z.0339@m.isct.ac.jp (G.Z.); ichikawa.kenta@tmd.ac.jp (K.I.); iitani.k.9009@m.isct.ac.jp (K.I.); nakajima@nakajimalab.org (Y.N.)

[2] Graduate School of Medical and Dental Sciences, Institute of Science Tokyo, 1-5-45 Yushima, Bunkyo-ku, Tokyo 113-8510, Japan; hd1216@outlook.com

* Correspondence: m.bdi@tmd.ac.jp

This PDF file includes:

Figures S1……………………………………………………………..S-2
Figures S2……………………………………………………………..S-3
Figures S3……………………………………………………………..S-4
Figures S4……………………………………………………………..S-5
Figures S5……………………………………………………………..S-6

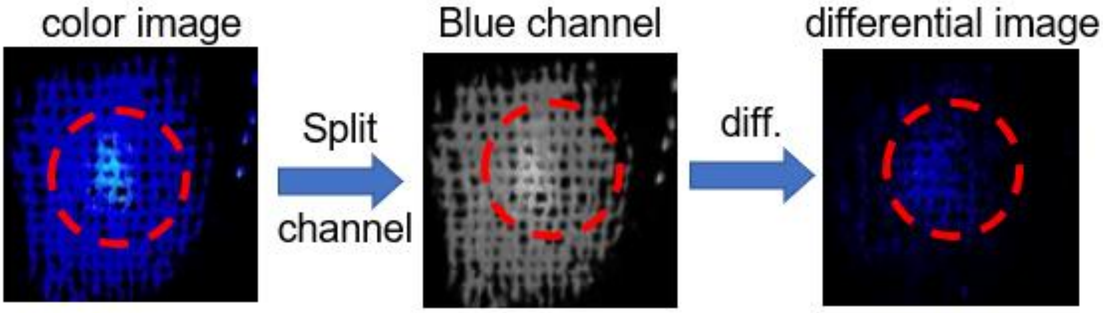

Figure S1. Representative fluorescence images with labeled key regions demonstrating the image processing workflow for NADH fluorescence quantification, including original color image, blue channel extraction, and differential image analysis. The central circular region (marked by red dashed line) indicates the gas delivery area on the ADH-immobilized membrane surface.

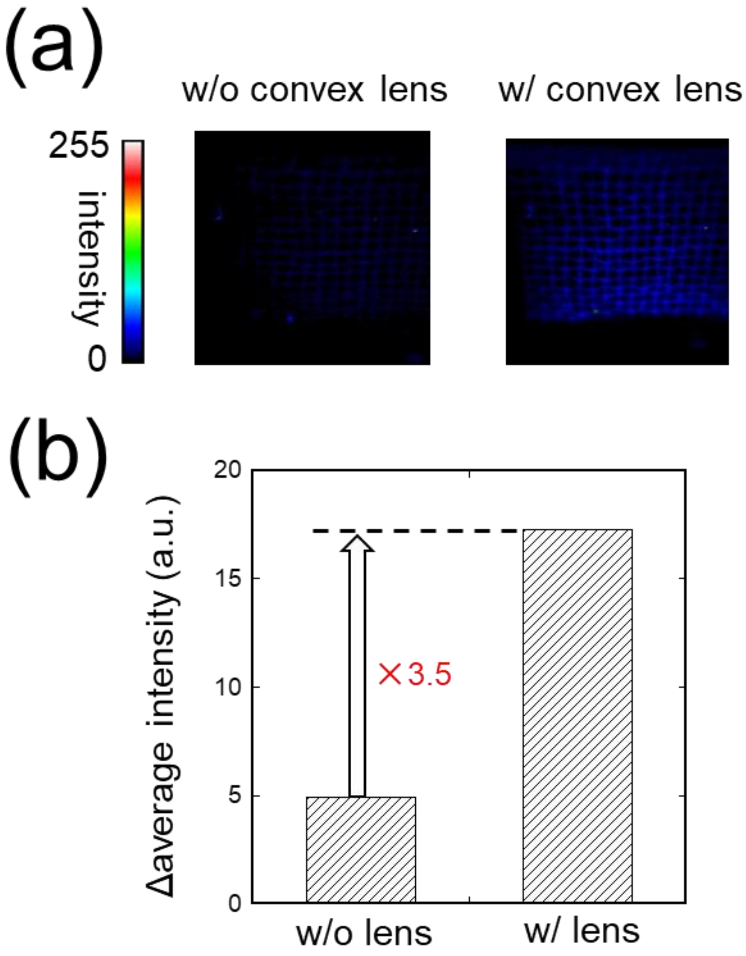

Figure S2. Effect of convex lens configuration on fluorescence detection efficiency. (a) Fluorescence images of a cotton mesh test piece excited and captured without (w/o lens) and with convex lenses (w/ lens). (b) Comparison of average fluorescence intensity.

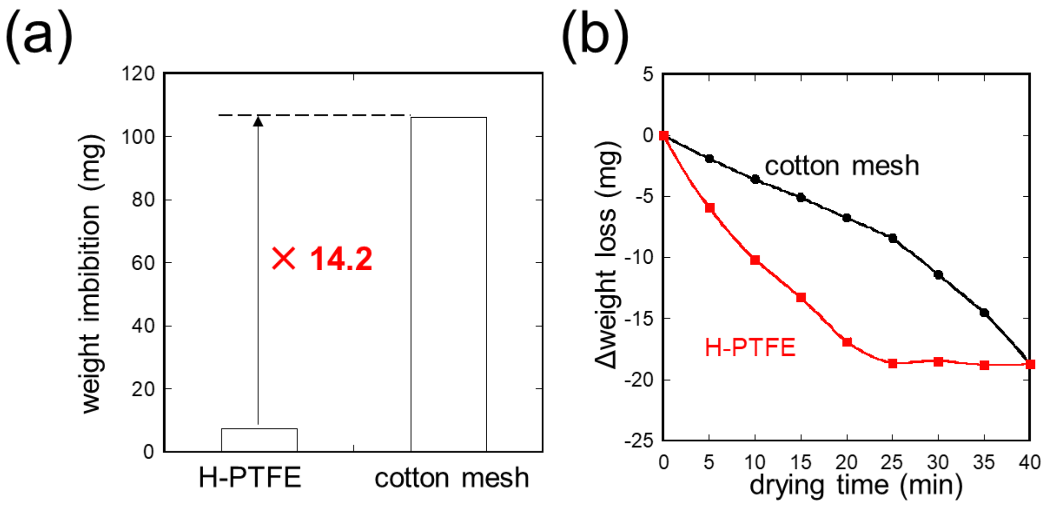

Figure S3. Water retention properties of H-PTFE membrane and cotton mesh. (a) Water imbibition capacity. (b) Water evaporation rate.

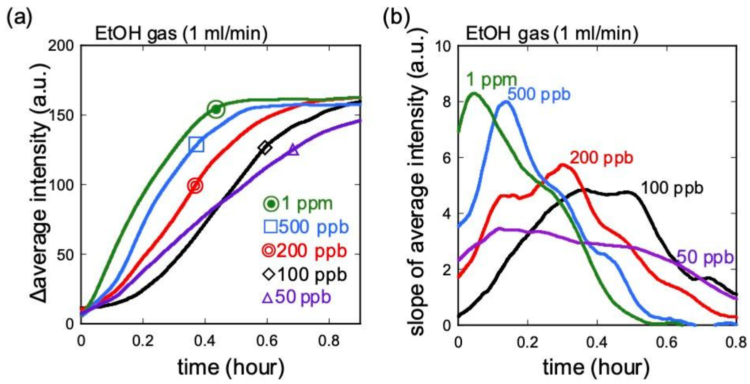

Figure S4. Long-term EtOH gas loading results. (a) Time courses of fluorescence intensity at EtOH concentrations of 50 ppb, 100 ppb, 200 ppb, 500 ppb, and 1 ppm. (b) Time courses of the slope of average fluorescence intensity.

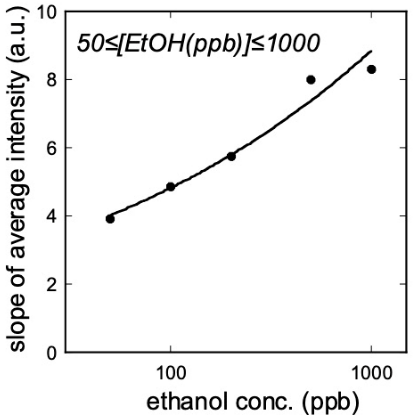

Figure S5. Calibration curve for long-term monitoring of ear-canal EtOH.
